# Fabrication of environmentally safe antifouling coatings using nano-MnO_2_/cellulose nanofiber composite with BED/GMA irradiated by electron beam

**DOI:** 10.1038/s41598-023-46559-1

**Published:** 2023-11-07

**Authors:** Madelyn N. Moawad, Khaled A. El-Damhogy, Mohamed Mohamady Ghobashy, Islam M. Radwan, Ahmed Nasr Alabssawy

**Affiliations:** 1 National Institute of Oceanography and Fisheries, NIOF, Cairo, Egypt; 2 Marine Science and Fishes Branch, Zoology Department, Faculty of Science, Al-Azhar University, Cairo, Egypt; 3 Radiation Research of Polymer Chemistry Department, National Center for Radiation Research and Technology (NCRRT), Egyptian Atomic Energy Authority (EAEA), Cairo, Egypt

**Keywords:** Environmental biotechnology, Environmental sciences, Nanoscience and technology

## Abstract

Marine biofouling, undesirable growth of organisms on submerged surfaces, poses significant challenges in various industries and marine applications. The development of environmentally safe antifouling coatings employing nano-MnO_2_/cellulose nanofiber (CNF) composite with bisphenol A epoxy diacrylate/glycidyl methacrylate (BED/GMA) irradiated by electron beam (T_1_) has been achieved in the current work. The physico-chemical characteristics of the fabricated coatings have been studied using Fourier transforms infrared spectroscopy, scanning electron microscope, water contact angle, and X-ray diffraction. The efficacy of T_1_ formulation and pure BED/GMA polymer (T_2_) in inhibiting biofouling formation was investigated in seawater of Alexandria Eastern Harbour by examining biofilm development morphologically and biochemically. In addition, regular analyses of seawater physicochemical parameters were conducted monthly throughout study. Results provide valuable information on coating performance as well as the complex interactions between coatings, biofilms, and environmental factors. The T_1_ formulation exhibited strong anti-fouling and anticorrosion properties over 2 months. However, after four months of immersion, all coated steel surfaces, including T_1_, T_2_, and T_0_, were heavily covered with macro-fouling, including tubeworms, barnacles, and algae. Biochemical analysis of extracellular polymeric substances (EPS) showed statistically significant variations in carbohydrates content between the coated surfaces. The T_1_ formulation showed decreased protein and carbohydrate content in EPS fractions after 14 days of immersion indicating less biofouling. Moreover, elemental analysis showed that carbon, oxygen, and iron were the predominant elements in the biofilm. Other elements such as sodium, silicon, chloride, and calcium were in lower concentrations. T_2_ and T_0_ surfaces revealed higher calcium levels and the appearance of sulphur peaks if compared with T_1_ surface. Diatoms and bacteria were detected on T_1_, T_2_, and T_0_ surfaces. The observed warming of seawater and nutrient-rich conditions were found to promote the growth of fouling organisms, emphasizing the importance of considering environmental factors in biofouling management strategies.

## Introduction

Biofouling is the process by which aquatic organisms, such as bacteria, algae, barnacles, and other marine organisms, colonize all surfaces exposed to water in the natural environment. It is a common phenomenon in marine and freshwater settings, including ships, underwater structures, pipelines, buoys, and marine equipment^1^. It involves four stages: initial attachment; microbial growth and biofilm formation; colonization by macroorganisms, and formation of a mature fouling community^2^. Van der Waals interactions during the biochemical conditioning stage result in the creation of a conditioning film that is mostly made up of organic molecules such as proteins, polysaccharides, and other biomolecules. Any surface in direct contact with seawater eventually goes through this process. Bacterial colonisation, or the second step, occurs when diatoms and bacteria begin to cling to the conditioning film and create a microbial biofilm. Bacteria and diatoms have specialized mechanisms for attaching to surfaces, such as producing adhesive extracellular substances or using appendages like pili. Unicellular eukaryotes, such as the spores of macroalgae, aggregate in the third phase. In the last stage, multicellular foulers like barnacles and mollusks are attached to the biofilm-covered surface. They often use adhesive secretions, specialized appendages, or physical mechanisms to establish a firm attachment. Numerous naturally occurring variables, such as salinity, temperature, light, or interactions between various creatures, are critical for this process to occur^3^. Understanding the complex interactions between these variables and the fouling process is essential for developing effective strategies to prevent or mitigate biofouling in marine environments. The adverse consequences of this unavoidable mechanism include increasing surface roughness, mass gain, and biocorrosion in a number of technical applications^3,4^. Particularly in the shipping sector, the accumulation of organisms increases drag resistance, which leads to up to 40% more fuel use, greenhouse gas emissions, and significantly higher transportation costs^3^. Traditionally, biocidal chemicals have been added to antifouling paint formulations to eliminate fouling organisms like algae, bacteria, and barnacles. The most powerful products in this regard, tributyltin (TBT)-based coatings, were created in the 1980s. It was demonstrated that they may greatly decrease fouling and, as a result, maintaining costs^3,5^. However, TBT was categorized as an environmentally harmful compound because of biocide's severe adverse effects on the marine environment^3^. Since the 2008 ban for TBT-based antifouling coatings, other substitutes have been established, such as Cu- and Zn-compounds. However, both the ocean and non-target organisms were seriously affected by these coatings^3^. Additionally, certain species, such as barnacles, have shown the ability to develop resistance to copper-based coatings, reducing their long-term effectiveness. Consequently, there is an urgent need to create new coatings that are antifouling-capable and environmentally friendly while also being mechanically stable. This includes exploring non-toxic and non-leaching coatings, such as fouling-release coatings that utilize low-surface energy materials to prevent fouling organisms from adhering to surfaces. Bio-inspired approaches, such as mimicking the slippery surfaces of certain marine organisms, are also being investigated^3^. Additionally, research is focused on developing novel biocidal compounds that are effective against fouling organisms but have reduced environmental impact. Recent advances in the field of inorganic nanomaterials may be crucial since the incorporation of nanoparticles can change the coating's rheological and adhesion characteristics, their antibacterial and generally biotoxic capabilities, offering a possible replacement for the conventional biocidal components used in antifouling coatings^6^. Being a well-known transition metal oxide with biological activity, manganese oxides have attracted a lot of investigation due to its outstanding structural diversity and distinctive chemical and physical characteristics^7^.

In contrast to other metallic oxide nanoparticles, nano-MnO_2_ can be synthesized immediately from readily accessible solvents. Many uses for nano-MnO_2_ have been described, and it has recently been discovered to possess powerful bactericidal abilities^8^. However, employing any metal oxides in powdered, particularly nanosized metallic oxides like manganese oxide, has various operational restrictions, including complications in the operating stages and the creation of dust contamination. Consequently, direct production of nanostructures on reinforced material is gaining popularity^7^. In recent decades, detailed investigations have concentrated on cellulose's function as a reinforcing material for inorganic nanostructures^9^. They are receiving tremendous attention as a support since they are generally accessible, inexpensive, renewable, and environmentally beneficial^7^. Due to all these distinct characteristics, nano-MnO_2_/cellulose composite may be used as fillers to produce effective coatings for antifouling applications. Radiation technique is popular method for crosslinked polymer approach^10,11^ in biomaterials^12^, water treatment membrane^13^, blend film polymer^14^, and renewable polymer^15–17^.

The primary goal of the current research was to develop environmentally friendly antifouling coatings using a nano-MnO_2_/cellulose nanofiber (CNF) composite with BED/GMA as matrix polymer, which was irradiated by an electron beam. The research addressed the need for alternative antifouling solutions that effectively prevent fouling while minimizing environmental impact. The fabricated nano-MnO_2_/CNF composite was examined comprehensively to assess its mechanical and chemical properties. Techniques such as Fourier transform infrared spectroscopy (FTIR), scanning electron microscopy (SEM), water contact angle (WCA), and X-ray diffraction (XRD) were employed to study the characteristics of the composite material. An experimental immersion test was conducted in the Eastern Harbour of Alexandria, Egypt to evaluate the antifouling performance of the BED/GMA-nano MnO_2_/CNF composite (T_1_) and the pure BED/GMA polymer (T_2_). Several assays were carried out to evaluate the morphological and biochemical alterations in the biofilm as well as the overall biofouling process during the study.

## Material and methods

### Materials

Sigma-Aldrich Chemie Gmbh Munich, Germany provided the bisphenol A epoxy diacrylate and glycidyl methacrylate. Potassium permanganate (KMnO_4_) and polyethylene glycol PEG-6000 were obtained from Beijing Chemical Reagent Company.

### Polyol method synthesis of MnO_2_/cellulose fiber nanocomposite

This section described process of the in-situ formation of manganese dioxide (MnO_2_) nanoparticles within cellulose nanofibers (CNF) using the polyol method synthesis. First, the preparation of CNFs involved the following steps: in a beaker containing 100 ml of distilled water, 4 g of cellulose and 0.04 g of polyethylene glycol (PEG) were mixed to create a suspension. To initiate the oxidation reaction, 10 mmol/l of sodium chlorite solution (NaClO) were added to the cellulose suspension. The mixture was then sonicated for 30 min. The oxidation reaction lasted for three hours at room temperature. The clean oxidized cellulose was filtered away from the suspension at the end of the three-hour reaction. The filtered cellulose oxide was suspended in water at 3 mg/ml. An ultrasonic generator with a probe diameter of 13 cm and an output power of 400 W was used to sonicate the cellulose oxide/water slurry for 10 min in an ice bath. After sonication, the mixture was centrifuged at 10,000 rpm for 10 min. The resulting CNFs were then pulverized. Second, the preparation of nano MnO_2_ involved the following steps: 12 mM of KMnO_4_ were dissolved in a 12% polyethylene glycol solution (PEG-6000). The solvent for the PEG-6000 solution is a mixture of ethanol and water in a volume ratio of 70/30, respectively. 0.03 g of CNF were immersed in the KMnO_4_/PEG solution and stirred for 30 min to ensure proper impregnation of CNF. The mixture of (KMnO_4_/PEG)/CNF were transferred into a sonication bath for 30 min. The (KMnO_4_/PEG)/CNF matrix was refluxed at 100 °C for 60 min. This step facilitates the reduction of KMnO_4_ to MnO_2_ through the reaction with polyethylene glycol. As a result, the color of the solution changes from faintly pink to brown, indicating the formation of MnO_2_/CNF nanocomposite. After refluxing, the resulting product was filtered to separate the nanocomposite from the solution. The filtered product was rinsed several times with distilled water and ethanol to remove residual impurities or unreacted chemicals. Finally, the washed nanocomposite was dried in an oven set at 50 °C for 1 h to remove any remaining solvent and moisture. This synthesis method enables the in situ formation of MnO_2_ nanoparticles within CNF, resulting in the fabrication of MnO_2_/CNF nanocomposites.

### Antifouling coatings fabrication and curing by EB irradiation

The process of formulating and preparing the coatings using different compositions of bisphenol A epoxy diacrylate (BED) and glycidyl methacrylate (GMA) along with the incorporation of MnO_2_/CNF nanoparticles was carried out as follows. Preparation of BED/GMA formulations: 1.5 ml, 2.5 ml, and 3.5 ml of BED were dissolved in 15 ml of acetone to create three formulations named 1G, 2G, and 3G, respectively. Similarly, 3.5 ml, 2.5 ml, and 1.5 ml of GMA were dissolved in 15 ml acetone to create the corresponding formulations. 0.2 wt% of MnO_2_/CNF nanoparticles were added to each BED/GMA formulation. The mixing was performed through a sonication process, which involves subjecting the compositions to ultrasonic waves to ensure the uniform distribution of nanoparticles within the formulations. The compositions were stirred continuously for 40 min to achieve homogenous mixes. Afterward, they were softly mixed for 15 min at 2000 rpm using a homogenizer at room temperature. This step further enhances the uniformity of the mixes. Thin layers of the two paint formulations were spread onto mild steel rectangular plates and tin, glass, and wood substrates. The coatings were applied using a film applicator, resulting in a thickness of approximately 100 μm. The coated plates were subjected to electron beam irradiation (EB) with a dose of 30 kGy. The irradiation process was carried out at room temperature using a beam accelerator with specific parameters: 90 kW power, 3 MeV energy, conveyor speed of 16 m/min (50HZ), 30 mA current, and a variable scan width of up to 90. This process aims to fabricate the antifouling coatings by incorporating MnO_2_/CNF nanoparticles into the BED/GMA formulations, then applying the coatings onto various substrates and subsequent electron beam irradiation. The specific parameters in the electron beam irradiation process comply with ASTM D 823-07 standards.

### Characterization of antifouling coating

#### Fourier transforms infrared spectroscopy (FTIR)

Chemical structures of all coated materials were measured using an FTIR spectrometer. Averaging 25 scans (400 to 4000 cm^−1^), infrared spectra were collected at 4 cm^−1^ resolution.

#### Scanning electron microscope (SEM)

A high-resolution JEOL scanning electron microscope JSM 5400 (Shimadzu Corporation, Japan) was used to examine the surface roughness of the coated samples undergoing EB curing. The photomicrograph orientation was maintained throughout the investigation. The surfaces were coated with a fine gold layer using a vacuum evaporation procedure (300–400 m). The photo was captured with a magnification of 10 μm.

#### Water contact angle (WCA)

The evaluation of surface tension and water contact angle (WCA) for the coated samples involved using techniques and equipment, including a goniometer. The surface tension of the coated samples was assessed by observing the behavior of water droplets on the material's surface. A goniometer is a device used to measure contact angles. The goniometer measures the contact angle between the solid surface and the droplet. This angle provides information about the surface's wettability and can be used to evaluate surface tension. The water shield refers to the ability of the coating to repel water. It is determined by measuring the water contact angle (WCA) of the water droplets on the surface. The WCA is typically measured at room temperature. The WCA values are obtained by measuring the same specimen multiple times (3 to 5 times) at different locations. Each measurement involves placing 100 μm water droplets on the surface and capturing the corresponding contact angles. The average WCA value is calculated by taking the average of the measured contact angles from the multiple measurements conducted on the same specimen. This averaging helps to obtain an expected value that reflects the surface tension characteristics of the coating. The water contact angle and its average value provide insights into the ability of the coatings to repel water, which is relevant to their antifouling properties.

### Material deployment studies

**Figure 1:**
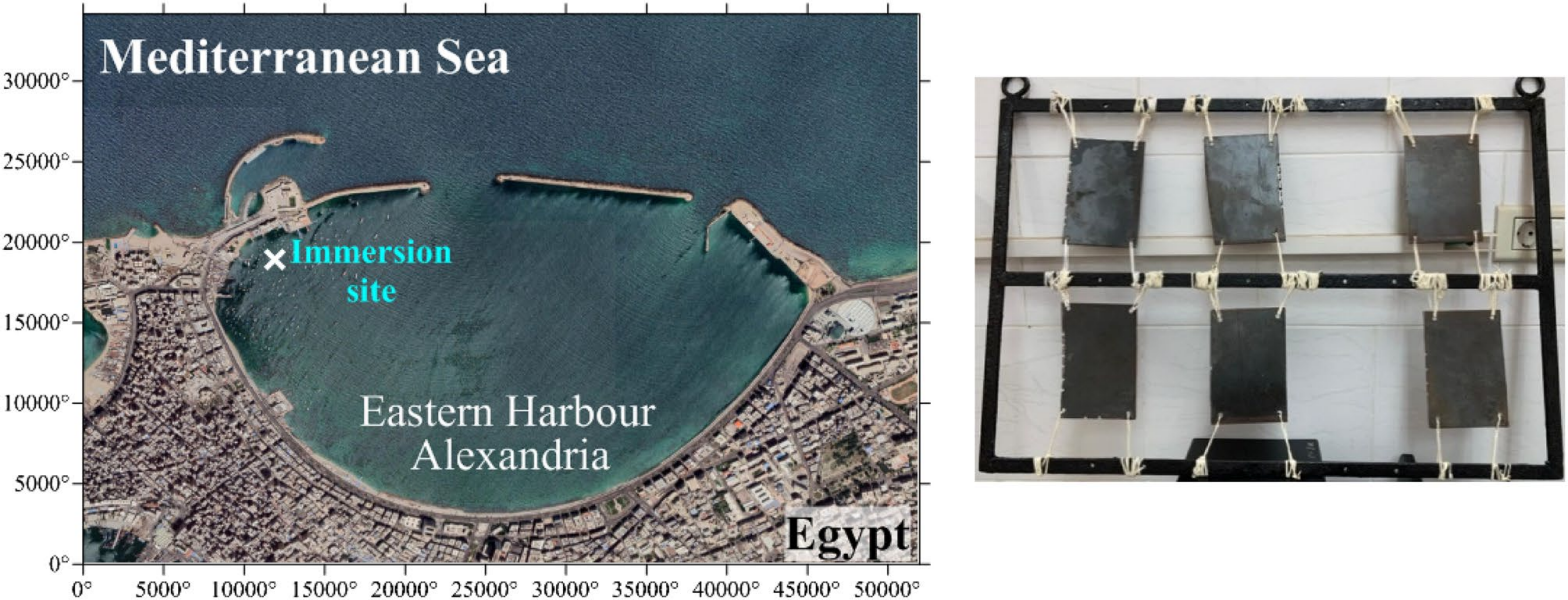
Immersion site of coated steel panels with two paint compositions (T_1_ and T_2_) along with the control panels (T_0_) in duplicate at Eastern Harbour.

The coated steel panels with two paint compositions, nano-MnO_2_/CNF incorporating BED/GMA (T_1_) and BED/GMA formulation (T_2_) were installed in pairs on 80 × 60 cm steel frame (Fig. 1). The control panels, which had no paint composition, were also installed alongside the coated panels. It served as a reference to compare the fouling resistance of the coated panels. The panels were attached to the steel frame using nylon thread. The trial occurred at the Eastern Harbour in Alexandria, Egypt, with the specific coordinates of 29° 53.12' E and 31° 12.67' N (Fig. 1). The steel frame holding the coated and control panels was immersed statically at a depth of 1 m. This depth ensured that the panels were submerged in water during the trial. The effectiveness of paint formulations (T_1_ and T_2_) as antifouling agents was investigated over four-month field trial, starting from July 1st, 2022 (Fig. A.1).

### Antifouling performance evaluation and characterization

#### Surface characterization (macroscopic and microscopic)

After immersion, the visual assessment of the coated panels was carried out using a digital camera. Monthly images were captured to record the progress of fouling on the panels throughout the research period. Additionally, adhered biomaterials and cells formed on coated plates were subjected to morphological characterization after two months of immersion and their elemental compositions were determined using a JEOL JSM-IT200 scanning electron microscope (SEM)-outfitted with an energy dispersive X-ray spectrometer (EDX). Following samples dehydration, metallization with gold was performed.

#### Mass assessment

The weight of the fouling organisms (FOW) that developed on the coated panels during the investigation was measured by weighing the steel panels before and after the study period. Samples were rinsed with distilled water and left to dry until the weights held steady. To determine the mass change, the weights of the steel panels before and after were deducted from one another.

#### Biochemical analysis of extracellular polymeric substance (EPS) forming biofilm

The steel panels were thoroughly cleaned with distilled water prior EPS extraction (protein and carbohydrate extraction). EPS extraction and characterization were performed at specific time points after 2, 7, and 14 days of immersion. These time points allowed for assessing EPS production and composition at different stages of fouling development. Sweeping samples were taken from the panel surfaces using a recovery swab with sample tube. The sample tubes were sonicated with 3 ml distilled water for 5 min to obtain maximum extraction of EPS from swab. Following sonication, particles that could be retained in the solution were removed using centrifugation at 4000 rpm for 15 min at room temperature. Protein and total carbohydrate levels in the EPS-containing supernatant were analyzed chemically in triplicate.

##### Protein determination

The Lowry technique was used to examine an extracted aliquot (0.5 ml) using bovine serum albumin as the reference material^18^.

##### Carbohydrate adsorption assay

Using a phenol–sulfuric acid reaction, carbohydrate measurement was performed on a 0.5 ml sample aliquot in accordance with DuBois et al.^19^.

### Physicochemical characteristics of seawater

Routine analyses of seawater's physicochemical properties were carried out throughout the study period. A thermometer measured the water's temperature on-site. Salinity and pH were measured on-site using a salinometer and a portable pH metre, respectively. A modified version of Winkler's method was used to quantify the concentrations of dissolved oxygen (DO)^20^. The most significant dissolved nutritional salts, including inorganic nitrogen, inorganic phosphate, and inorganic silicate, were measured calorimetrically using a double-beam spectrophotometer of Jenway 6305 brand in accordance with the instructions provided by Parsons et al.^21^.

### Statistical analyses

The statistical analysis of the collected data was performed using Microsoft Excel-2010 software.

## Results and discussion

### Physicochemical characterization of MnO_2_ nanoparticles and EB cured coating films of BED/GMA

**Figure 2:**
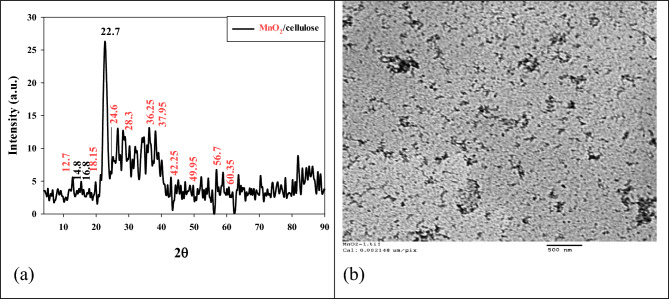
The XRD (**a**) and TEM image (**b**) of MnO_2_/CNF nanocomposite.

In Fig. 2a, the X-ray diffraction (XRD) pattern shows characteristic peaks indicating the presence of CNF and MnO_2_ nanoparticles. The peak located at 2θ = 22.7° corresponds to the (120) plane of the cellulose nanofibers, which exhibits high intensity. Two additional peaks at 2θ = 4.8° and 16.6° correspond to cellulose's type I crystal structure^22^. The XRD pattern also shows distinct peaks attributed to MnO_2_ nanoparticles. The peaks at 2θ = 12.7°, 18.15°, 28.3°, 37.5°, 42.25°, 49.95°, 56.7°, and 60.3° correspond to the (110), (200), (310), (211), (301), (411), (600), and (521) lattice planes of α-MnO_2_, respectively. These peaks are consistent with the α-MnO_2_ standard data from the JCPDS card (PDF file no. 44–0141)^23^. To determine the mean crystal size (D) of the α-MnO_2_ nanoparticles, the Scherrer equation D = Kλ/βcosθ was used, where K is a constant, λ is the X-ray wavelength, β is the full width at half maximum (FWHM) of the diffraction peak, and θ is the diffraction angle. By measuring the sharpest diffraction peak of α-MnO_2_ (211), the calculated crystal diameter of α-MnO_2_ was 35 nm. Figure 2b presents the morphologies of the α-MnO_2_ nanoparticles as observed by transmission electron microscopy (TEM). This imaging technique provides a detailed view of the nanoparticle structures and confirms their size and shape. The XRD analysis and TEM imaging provide valuable information about the crystalline structure and morphology of the nano-MnO_2_/CNF composite, demonstrating the successful synthesis and characterization of the MnO_2_ nanoparticles within the CNFs.

**Figure 3:**
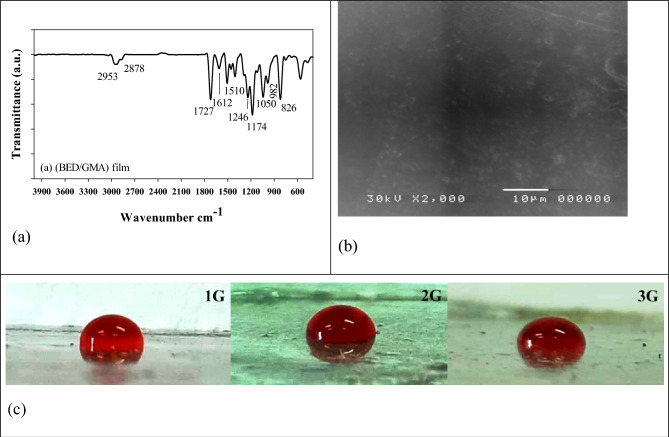
FTIR (**a**), surface morphology (**b**), and the surface wettability properties (**c**) of the EB cured (BED/GMA) film.

Figure 3a shows the Fourier-transform infrared (FTIR) spectroscopy analysis of the electron beam-cured (BED/GMA) film. The spectrum exhibits several characteristic peaks related to specific functional groups in the cured film. One prominent peak appears at 1732 cm^−1^, corresponding to the carbonyl group (C=O) stretching vibration. This signal indicates the presence of acrylate and epoxy groups in the BED/GMA polymer^24^. The FTIR peaks at 2860 cm^−1^ and 2930 cm^−1^ represent the symmetric and asymmetric stretching vibrations of aliphatic (C–H) bonds, respectively. These signals are characteristic of the carbon-hydrogen bonds in the cured film. The FTIR peak at 1612 cm^−1^ indicates the aliphatic unsaturated bonds (C=C) presence. This peak confirms the retention of unsaturated groups in the cured sample. Notably, the absence of a signal in the range of 3200–3400 cm^−1^ indicates the absence of the hydroxyl group (–OH), suggesting that the hydrophilic nature of the cured (BED/GMA) film is reduced. Furthermore, the FTIR spectrum shows peaks at 826 cm^−1^ and 1246 cm^−1^, corresponding to the deformation vibrations of the C–H bonds in the epoxy ring structure. The FTIR analysis provides insights into the chemical composition and functional groups in the electron beam-cured (BED/GMA) film, confirming the presence of carbonyl groups, aliphatic bonds, unsaturated bonds, and epoxy ring structures.

Figure 3b displays the surface morphology of the electron beam-cured (BED/GMA) film after coating with gold. The fractured surface of the film reveals certain features that contribute to its adhesive properties. One notable observation is the clear indication of handedness, which refers to the orientation or alignment of the film's molecular structure. This handedness is likely responsible for the improved adhesive properties of the film, as it promotes stronger interactions between the coating and the substrate. Pores, cavities, and aggregations zones in the fractured surfaces are significantly minimal. This indicates that the film has a relatively smooth and homogeneous surface, with fewer irregularities or defects. The reduced presence of such features can contribute to enhanced coating inhibition by preventing water molecules or dissolved ions from adhering to the surface of the substrate. Finally, the surface morphology analysis suggests that the electron beam-cured (BED/GMA) film exhibits favorable adhesive properties, with a relatively smooth and defect-free surface. These characteristics contribute to its effectiveness as a waterproof coating by preventing the adherence of water molecules and dissolved ions to the substrate's surface.

Figure 3c illustrates the water contact angle (WCA) fluctuation on the steel surface coated with BED/GMA film compositions at various concentrations. The WCA values serve as an indicator of the hydrophobicity of the coated films. Comparing the different formulations, it can be observed that as the concentration of GMA increases from G1 to G3, the WCA levels decrease from 69° to 58° and then to 31°. This indicates that the hydrophobicity of the coated films increases with higher levels of GMA. The homogeneity and excellent dispersion of BED and GMA in the film contribute to the formation of a highly hydrophobic surface when the concentration of GMA is increased.

On the other hand, it is noted that the WCA values decrease as the concentration of BED increases. This implies that higher concentrations of BED lead to a reduction in the hydrophobicity of the coated films. Therefore, the G1 formulation, with a lower BED concentration, is favored as it provides an extremely water-resistant surface. The WCA measurements demonstrate the influence of BED/GMA concentrations on the hydrophobicity of the coated steel surfaces. The findings suggest that the G1 formulation achieves a highly water-resistant surface with an optimal combination of BED and GMA.

### Antifouling performance

A number of tests were used to assess the coated materials efficacy as fouling inhibitors. These tests evaluated the biofouling process as well as the morphological and biochemical alterations in the biofilm.

#### Visual assessment

**Figure 4:**
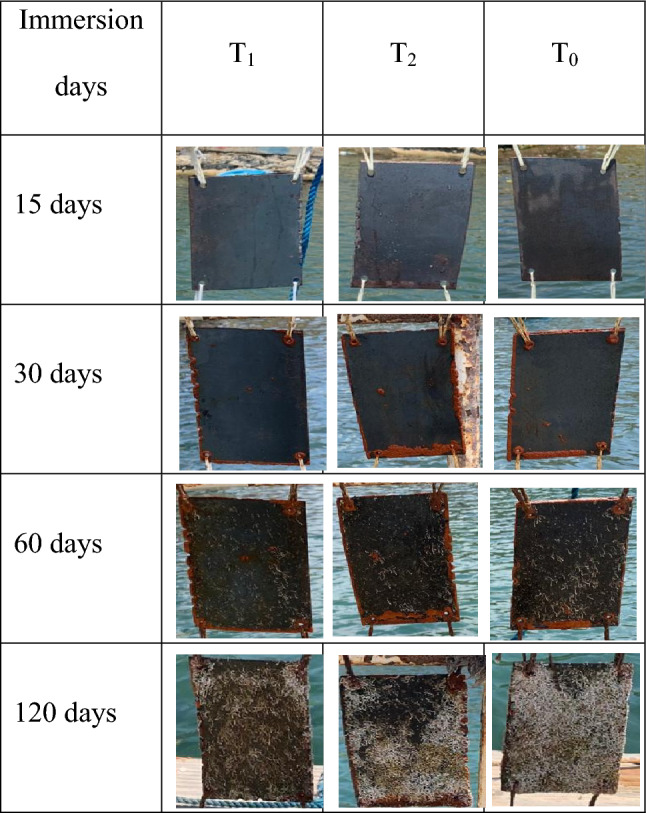
The progression of fouling species on steel panels during immersion period in the Eastern Harbour.

The results presented in Fig. 4 indicate the development of biofouling populations on the metal panels over four months of submergence in the Eastern Harbour. All samples had no fouling on third day. After 14 days of immersion, a visual assessment revealed that T_1_ and T_2_ coated panels remained free of corrosion and fouling organisms, while localized corrosion and deposits of a thick black film were observed on T_0_ surface. These findings suggest that T_1_ and T_2_ formulations effectively prevented the attachment and growth of fouling organisms (Fig. 4).

After a month of immersion, T_1_ and T_2_ coated panels remained in good condition, with only a few instances of *Calyptotheca alexandriensis*, an introduced species in the Eastern Harbour^25^, near the holes and edges. The fouling organisms appeared in the following order: T_1_˂ T_2_ ≤ T_0_, indicating that T_1_ formulation had the least contaminated surfaces compared to T_2_ and T_0_ formulations.

After 60 days of exposure, T_1_ coated plates with only a few tubeworms and *C. alexandriensis* had the least contaminated surface, while T_2_ and T_0_ panels were heavily covered with tubeworms. Two months later, tubeworms totally covered T_2_ and T_0_ steel panels in comparison to T_1_ steel panels. However, all panels had slight presence of barnacles and green algae. Summer changes in water quality may be associated to the development of barnacles, green algae, and an increase in the number of tubeworms on the surfaces of steel panels. According to the findings reported by Li and colleagues^26^, warmth (29 °C) enhanced the quantity, mineral content, and predator-resistance of tubeworms. Therefore, the warming of the seawater during the summer months may contribute to the increased adhesion strength and the growth of tubeworms, demanding the adoption of more strong antifouling or removal approaches.

Generally, the T_1_ formulation demonstrated strong anti-fouling and anticorrosion performance over a 2-month period. However, after four months of deployment, the surface of the experimental panels (T_1_, T_2_, and T_0_) was heavily covered with macro-fouling such as tubeworms, barnacles, and algae.

#### Extracellular polymeric substance characterization

The extracellular polymeric substances (EPS) released by biofilm-forming bacteria were extracted and analyzed for the two main EPS constituents, protein and total carbohydrate (Table 1). Lower levels of proteins and carbohydrates indicate less biofouling. The protein and carbohydrate content of EPS fractions developed on T_1_, T_2_, and T_0_ varied during the first 14 days of the investigation according to one-way ANOVA analysis (Table A.1–2). The form of carbohydrates present in EPS is largely influenced by the microbial community that makes up the biofilms^27^.

**Table 1:** The protein and carbohydrate content of EPS fractions developed on T_1_, T_2_, and T_0_ during the first 14 days.

Coated panels	Proteins (μg/surface)			Carbohydrates (μg/surface)		
	2 days	7 days	14 days	2 days	7 days	14 days
T_1_	292	172	163	1141	1005	476
T_2_	200	215	194	245	1666	1747
T_0_	270	204	223	452	1667	1734

**Figure 5:**
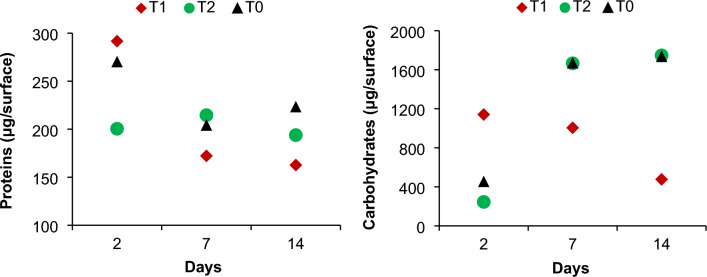
The two main EPS components in biofilm; proteins and total carbohydrates.

EPS on the steel plates coated with T_2_ formulation had the lowest protein and carbohydrate content (200 and 245 μg, respectively) after two days of seawater exposure. The T_1_-coated panel had the lowest protein and carbohydrates content after 7 and 14 days of immersion (Table 1 and Fig. 5). These results suggest that T_1_ formulation was particularly effective in reducing the accumulation of proteins and carbohydrates indicating lower levels of biofouling. This is in line with the findings of visual examination performed after two months of exposure, which revealed that T_1_ coated plates had the least contaminated surface, with just a few tubeworms and *C. alexandriensis* present, compared to T_2_ and T_0_ panels.

Furthermore, convenient quantitative method for assessing biofilm adherence to any platform is to measure the weight of fouling organisms^28^. Tables A.3–4 show the weight of the fouling organisms on the examined panels and the statistical variations in biofouling weights using one-way ANOVA, respectively. After the research period, T_1_-coated panel had the lowest weight of the fouling organism, followed by T_2_ and T_0_-coated panels. These findings were verified by visual and photographic examinations. The results indicate that the T_1_ formulation exhibited superior performance in reducing protein and carbohydrate content in EPS fractions and minimizing the weight of fouling organisms. This suggests that T_1_ effectively inhibits biofilm formation and growth, making it a good coating formulation for antifouling applications.

#### Biofilm architecture and elemental compositions (SEM–EDX)

**Figure 6:**
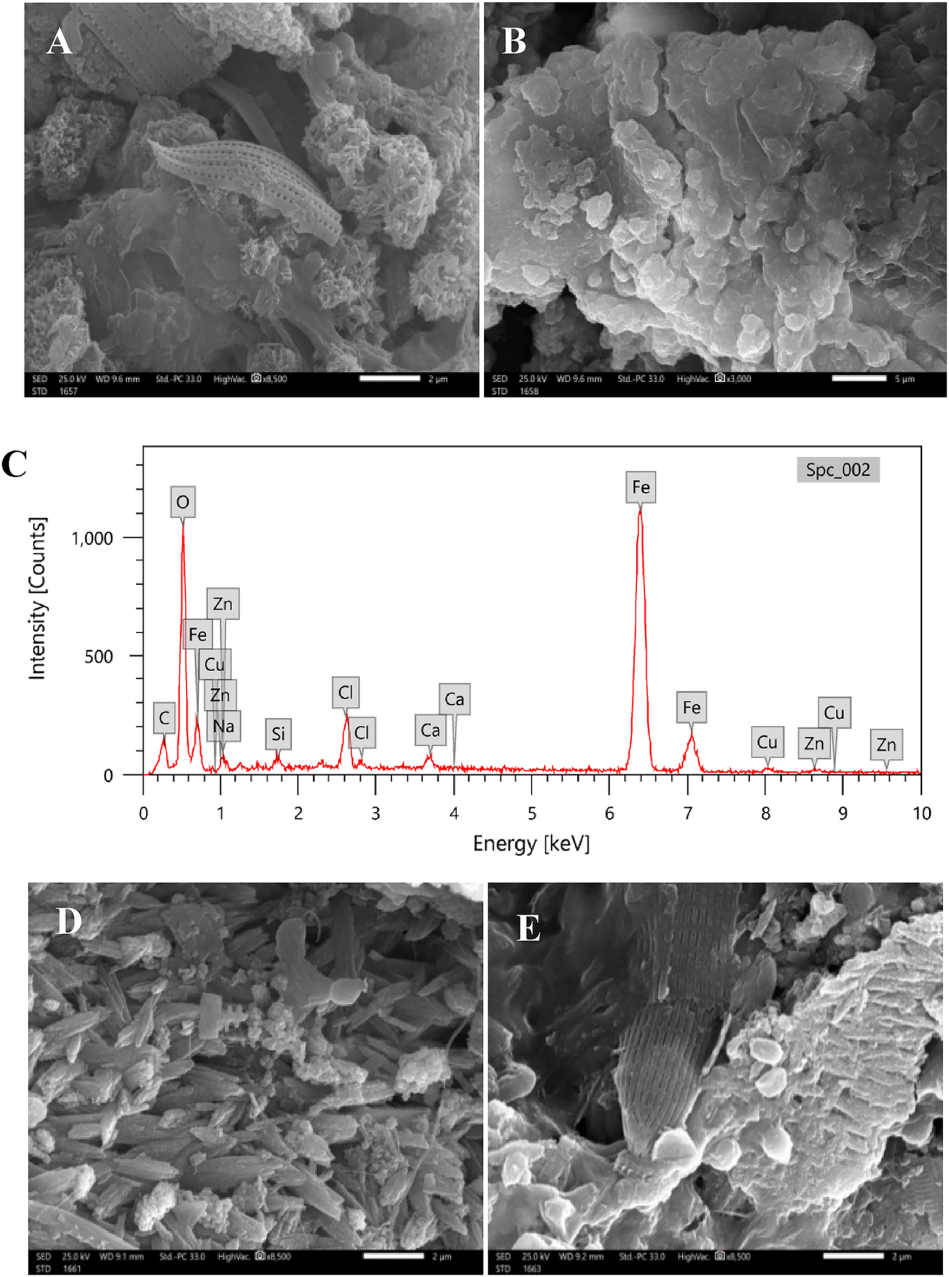

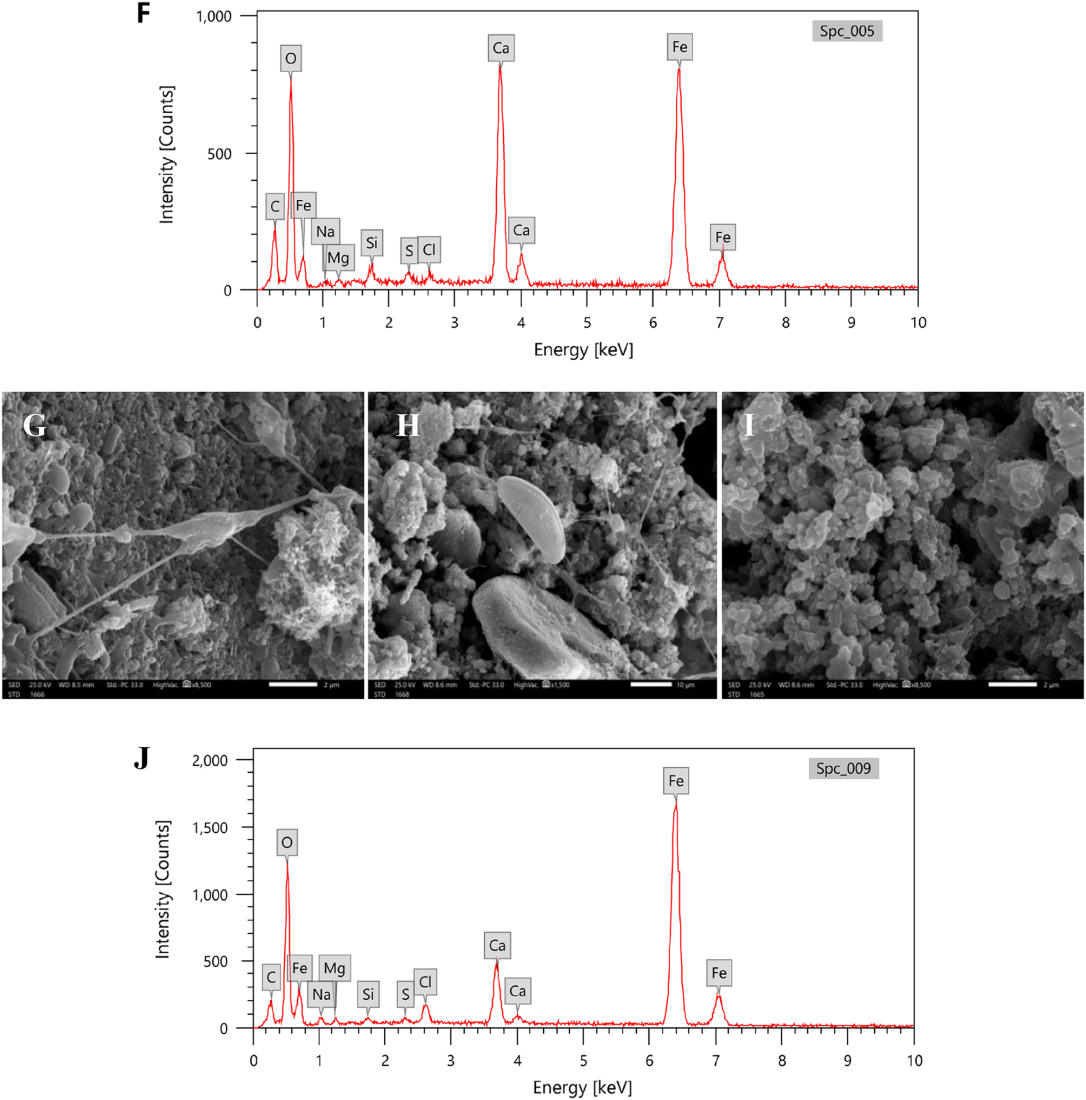
Scanning electron microscopy SEM and EDX analysis of biofilm layer on different treated steel panels, T_1_ (**A–C**), T_2_ (**D–F**), and T_0_ (**G–J**).

After two months of immersion, adhered biomaterials and cells produced on coated plates were subjected to morphological characterization, and their elemental compositions were identified using SEM-equipped with EDX. The weight and atomic percentage of nine elements, carbon, oxygen, iron, sodium, silicon, chloride, calcium, copper, and zinc, were detected by EDX analysis of biomaterial on T_1_ steel surface (Fig. 6C). The C, O, and Fe had the highest weight percentages (nearly 90%), whereas Na, Si, Cl, Ca, Cu, and Zn did not surpass 10%. In present study, biofilm on T_1_ surface acquired local amounts of heavy metal ions including Cu and Zn since many bacteria form exopolymeric acidic polysaccharides that may bound and adsorb metals from seawater^29^.

In contrast, the EDX spectra of T_2_ and T_0_ surfaces revealed higher calcium levels, particularly in T_2_, and the appearance of sulphur and magnesium peaks (Fig. 6F, J). Peaks of Cu and Zn were no longer observed. The presence of iron and sulphur in EDX spectra suggests the formation of iron sulphides, which are known to be produced by sulphate-reducing bacteria (SRB) in corrosive environments. The biofilm development on metal surfaces in marine environment is considered to be distinct from that on other surfaces, since iron and its numerous oxidative states available on steel offer some bacterial and archaeal populations with an attractive metabolic substrate. As a result, colonies of iron processing bacteria are frequently detected on metallic surfaces in marine environment, where they may cycle Fe (II) and Fe (III). Additionally, the hypoxic conditions necessary for sulfur cycling also promote the growth of SRB^30^, which forms the corrosive iron sulphides seen on T_2_ and T_0_ surfaces.

The topology of biofilms may be examined in considerable detail using scanning electron microscopy^30^. A marine biofilm colony can comprise a wide range of species, including fungi (12–18 μm), yeast (3–5 μm), bacteria (1 μm), algae (∼25 μm), and cilitae (> 200 μm). This is crucial considering the roughness of the substrata that tubeworms, barancles, or seaweeds will grow on the surface^31^.

Individual bacteria could not be identified in the gelatinous biofilm that covered the T_1_ mild steel surface (Fig. 6B), but some diatoms were entangled in the mucilaginous matrix of the T_1_ and T_2_ treated steel surface as observed in Fig. 6A, E.

The raised Ca peak in the EDX was verified by the presence of a scale-like structure on the T_2_ surface (Fig. 6D), indicating the presence of calcifying organisms, notably tubeworms in the current biofouling community. Various microbial species were found embedded in a thicker biofilm on the T_0_ surface (Fig. 6 G, H, and I), where the extracellular polymer enclosing the cells in Fig. 6G, H may be condensed to fine stretched filaments. Furthermore, the grouped bacterial cells seem to be partly coated with biofilm under corrosion layers suggesting the involvement of bacteria, such as SRB, in localized corrosion processes (Fig. 6G). The corrosion layers appear as rod-shaped particles with a rough, hazy surface under a scanning electron microscope, suggesting that metal sulphides have contained bacterial cells^32^, when they are in contact with metal substrate^29^.

In general, various microbial populations developed on the three mild steel panels. Diatoms were observed on T_1_, T_2_, and T_0_ mild steel panel. The biofilm's elemental composition on the three steel surfaces reveals the existence of diatom frustules with a silicon peak. Biofilms on T_0_ coated steel surfaces seemed to be more versatile, with diatom frustules and numerous microbial communities, followed by T_2_ steel substrate. Previous studies have also reported the colonization of diatoms and bacteria as first and the most abundant colonizers on various antifouling coatings substrates^33–37^.

In summary, the EDX analysis and SEM observations provide valuable information about the elemental composition and morphology of the biofilm on the steel surfaces. The presence of organic matter, iron compounds, heavy metal ions, and the colonization of diatoms and other microbial populations contribute to the complexity of the biofilm community and the biofouling process on the coated steel surfaces.

### Environmental parameters

It is important to take into account the surrounding environmental factors while conducting marine biofilm investigations, especially in biofouling management because it has been shown that both biofilms and macrofoulers may be impacted by these factors^38^. Furthermore, in order to create effective antifouling techniques, it is important to understand how fouling organisms will react to environmental stresses triggered by global warming, such as temperature, pH, and salinity^26^. However, it is challenging to compare the antifouling performance of the marine paints due to the variations in environmental conditions at different immersion locations. Despite the fact that little studies have recorded the environmental descriptions of the immersion sites^39^, it is important to consider the physicochemistry of a substratum, as well as environmental factors like nutrient levels, pH, DO, light accessibility, sample depth, and temperature, as they influence the biofilm development process in the marine environment^38^. Therefore, monthly surface water samples were obtained from Eastern Harbour during the immersion period, which extended from July to October 2022 representing the summer season (Table 2). Water temperature of 29.7 °C (27 to 32.1 °C), salinity of 37.3 (36.7–37.8), pH of (7.4–7.9), and DO of 2.8 mg/l (1.4–4.0 mg/l) are the determined averages and ranges for these variables. Microorganism adherence and growth rate on steel surfaces are influenced by temperature; as temperature rises, the growth rate accelerated. Microorganism reproduction starts in the spring and lasts through the summer^40^. Additionally, the warming of the seawater (~ 30 °C) observed in this study is anticipated to increase the adhesion strength of the primary biofouling organisms, such as tubeworms^26^. The total inorganic nitrogen and phosphate maintained high concentrations of 18.9 μM (16.8 to 26.3 μM) and 0.58 μM (0.53–0.62 μM), respectively. Biofilms grow quickly in nutrient-rich water^40^. These environmental factors is essential for accurately interpreting and evaluating the performance of antifouling coatings and understanding the dynamics of biofilm development and fouling processes in marine environments. By incorporating environmental data into research, scientists can better assess the efficacy of antifouling techniques and develop strategies that account for the effects of global warming and other environmental stressors.

**Table 2:** Monthly hydrographical parameters of surface water samples obtained from Eastern Harbour during the immersion period representing the summer season.

Time	Water temperature (°C)	pH	Salinity	DO (mg/l)	Nutrient salts (μM)					
					Ammonia	Nitrite	Nitrate	Total inorganic nitrogen	Phosphate	Silicate
0 time	30.9	7.4	37.8	2.8	22.5	1.2	2.6	26.3	0.58	8.4
30 days	30.8	7.9	36.7	1.9	13.1	1.2	2.6	17.0	0.62	6.4
60 days	32.1	7.6	–	1.4	15.2	1.2	1.1	17.5	0.62	6.2
90 days	27.6	7.8	37.3	3.8	10.0	1.4	5.4	16.8	0.53	6.4
120 days	27.0	7.7	37.5	4.0	8.8	1.3	6.8	16.8	0.53	4.7
Average	29.7		37.3	2.8	13.9	1.2	3.7	18.9	0.58	6.4

## Conclusion

The current work has developed environmentally safe antifouling coatings using BED/GMA-nano MnO_2_/CNF composites irradiated by electron beam (T_1_). Several coated film characteristics were measured to evaluate homogeneity and the curing process. It was found that formulae comprising 1.5 ml of BED and 3.5 ml GME (G1) greatly enhanced most of the coated film characteristics, including toughness, swelling/gel, resistance to abrasion, adherence, and chemical stability. The T_1_ formulation and pure BED/GMA polymer (T_2_) were also examined as an antifouling agent by exposing coated steel panels to seawater environment for a specific duration. From the results of antifouling, the T_1_ surface could resist biofouling and corrosion development over two months despite the higher temperature levels and nutrient enrichment found during the study period that encouraged microbe adherence and macroorganism settling. However, after four months of immersion, all coated steel surfaces, including T_1_, T_2_, and T_0_, were heavily covered with macro-fouling, including tubeworms, barnacles, and algae. This suggests long-term fouling resistance may require more robust antifouling strategies or removal approaches. The observed warming of seawater and nutrient-rich conditions were found to promote the growth of fouling organisms, emphasizing the importance of considering environmental factors in biofouling management strategies. Overall, this study highlights the complex interactions between coatings, biofilms, and environmental factors in marine environments. It underscores the need for continuous research and development of effective antifouling techniques that can withstand the challenges posed by biofouling and environmental stresses. By understanding the dynamics of biofilm development and considering the specific environmental conditions at different immersion sites, scientists can strive to develop more sustainable and efficient approaches for biofouling management in marine applications.

### Supplementary Information

Supplementary Information.

## Data Availability

All data analyzed during this study are included in this published article and supplementary file.
