# Supplementary material for: Fabrication of environmentally safe antifouling coatings using nano-MnO2/cellulose nanofiber composite with BED/GMA irradiated by electron beam
Source: Sci Rep. 2023 Nov 7;13:19289. doi: 10.1038/s41598-023-46559-1 (PMC10630369; doi:10.1038/s41598-023-46559-1)
Supplement: Supplementary file 1 — Supplementary Information. [file 41598_2023_46559_MOESM1_ESM.docx]

**Fabrication of environmentally safe antifouling coatings using nano-MnO_2_/cellulose nanofiber composite with BED/GMA irradiated by electron beam**

Madelyn N. Moawad ^a*^, Khaled A. EL-Damhogy^b^, Mohamed Mohamady Ghobashy ^c*^, Islam M. Radwan ^a^, Ahmed Nasr Alabssawy ^b^

^a^National Institute of Oceanography and Fisheries (NIOF), Cairo, Egypt.

^b^Marine Science and Fishes Branch, Zoology Department, Faculty of Science, Al-Azhar University, Cairo, Egypt.

^c^Radiation Research of Polymer Chemistry Department, National Center for Radiation Research and Technology (NCRRT), Egyptian Atomic Energy Authority (EAEA), Cairo, Egypt.

*Corresponding author E- mail: [madelynattia@gmail.com](mailto:madelynattia@gmail.com)

*Corresponding author E- mail: [Mohamed.ghobashy@eaea.org.eg](mailto:Mohamed.ghobashy@eaea.org.eg)

**Supplementary material**

**
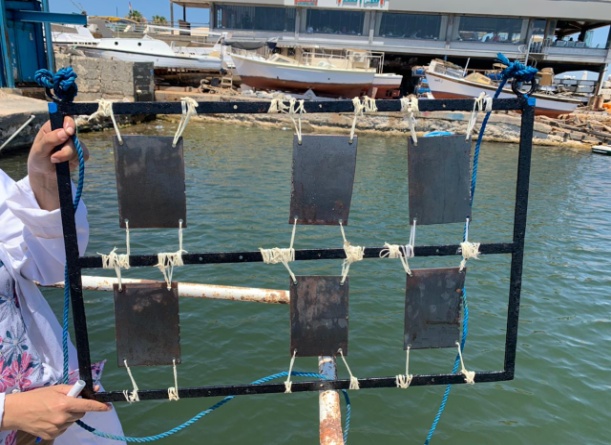

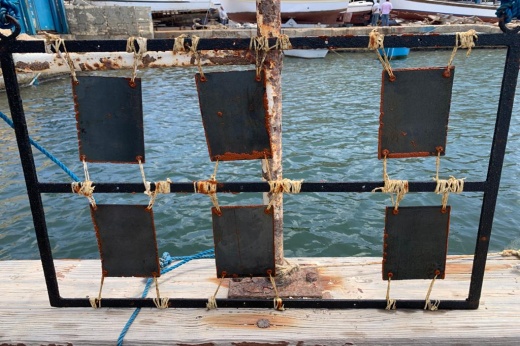
**

**
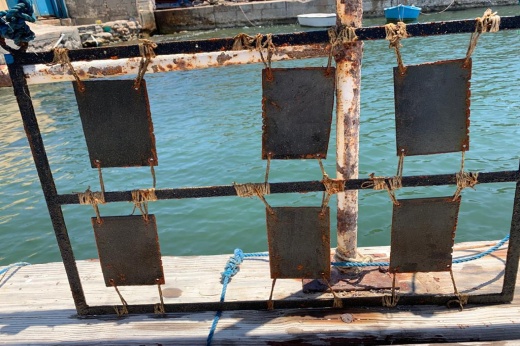

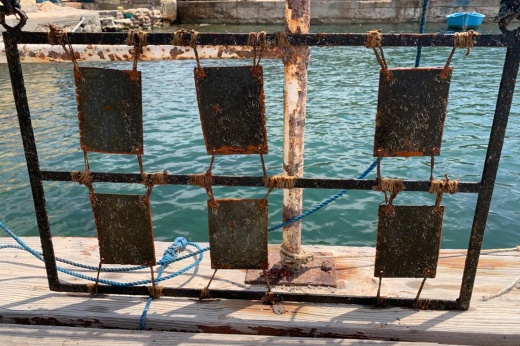
**

**Fig. A.1** The development of fouling organisms on steel panels during their immersion in the Eastern Harbour

Table A.1 One-way ANOVA analysis for protein and carbohydrate content of EPS fractions developed on steel panels after 2,7, and 14 days of immersion

| EPS content | *F* | P-value |
| --- | --- | --- |
|  |  |  |
| Proteins | 3.40 | 0.046* |
| Carbohydrates | 9.65 | 0.001* |

*Significant at the 0.05 level

Table A.2 One-way ANOVA analysis for protein and carbohydrate content of EPS fractions developed on coated steel panels (T_1_ and T_2_) and negative control (T_0_) during the first 14 days

| Immersion days | Proteins | | Carbohydrates | |
| --- | --- | --- | --- | --- |
|  | *F* | P-value | *F* | P-value |
| 2 days | 1.343 | 0.309 | 15.696 | 0.001* |
| 7 days | 0.980 | 0.412 | 10.680 | 0.004* |
| 14 days | 1.194 | 0.347 | 248.697 | 0.000* |

*Significant at the 0.05 level

Table A.3 Average fouling weight (FOW) formed on the panels (g/panel)±SD at the end of the experiment

| Extracts | Panels | | Average FOW (g**)** |
| --- | --- | --- | --- |
|  | 1^st^ row | 2^nd^ row |  |
| T_1_ | 182.9 | 181.02 | 181.96±0.94 |
| T_2_ | 260.35 | 231.35 | 245.85±14.50 |
| T_0_ | 277.88 | 292.73 | 285.31±7.43 |

Table A.4 One-way ANOVA analysis for weight of fouling organisms of different coating formulations (T_1_ and T_2_) and negative control (T_0_) at the end of the experiment

| Weight of fouling organisms (g/panel) | *F* | P-value |
| --- | --- | --- |
|  | 30.644 | 0.010* |

*Significant at the 0.05 level
